# Evaluating Eye Drop Instillation Technique and Its Determinants in Glaucoma Patients

**DOI:** 10.1155/2018/1376020

**Published:** 2018-04-08

**Authors:** Xinbo Gao, Qiongman Yang, Wenmin Huang, Tingting Chen, Chengguo Zuo, Xinyan Li, Wuyou Gao, Huiming Xiao

**Affiliations:** State Key Laboratory of Ophthalmology, Zhongshan Ophthalmic Center, Sun Yat-sen University, Guangzhou 510060, China

## Abstract

**Aim:**

To evaluate eye drop instillation technique and to explore its determinants in glaucoma patients.

**Methods:**

One hundred and thirteen patients diagnosed with glaucoma and self-administering topical antiglaucoma eye drops for at least 1 month were evaluated. All patients instilled artificial tear solution in one eye as they would do at home. The whole process was evaluated by two study staff. A comprehensive score system associated with eye drop instillation techniques was used to quantify the instillation technique and explore its determinants such as demographic and clinical characteristics.

**Results:**

Half of the patients (48.67%) finished the administration of eye drop on first attempt.1.7 eye drops were squeezed out on average. 43 patients (37.17%) got contact with ocular surface or adnexa. Only 19.7% patients had eye drop instillation techniques being defined as well. 11 patients (9.7%) had prior instruction regarding using eye drops, while only 4 patients knew to occlude the tear duct by pressing the dacryocyst area. Older age and worse visual acuity were found to be independent risk factors for worse instillation technique.

**Conclusions:**

Eye drop instillation technique in glaucoma patients deserves great attention from eye care practitioners during their lifelong follow-up, especially those aged older and have worse visual acuity.

## 1. Introduction

Glaucoma is a chronic eye disease causing blindness in millions of people worldwide [1]. Ocular hypotensive eye drops are the most common treatment for lowering the intraocular pressure, which until now is the only proved way to slow the progression of the disease. During lifelong follow-up, a daily, correct administration by the patient is required. However, it is reported nearly half of the patients with glaucoma could not use the eye drops properly [2].

Unlike oral medicines, eye drops require patients to use proper technique for successful medication administration. This requires not only instilling a single drop accurately into the conjunctiva of the eye but also without contacting eye drop container with the ocular surface or adnexa. It is reported more than half of the patients omitted 10% of doses and 15% of patients omitted half doses [3]. Poor eye drop instillation in adherence not only leads to reduced treatment effectiveness but also increases costs in such chronic disease. Systemic side effects, infection, or trauma can also be induced due to overdose or contacting the eye drop container with the eye [4]. Accordingly, this study aimed to explore the status of patients in busy clinical setting of a developing country and to evaluate the determinants of the drop instillation skill.

## 2. Methods

A cross-sectional design was used and 113 patients with glaucoma were enrolled in this study between August 1, 2016 and December 30, 2016. Nine patients who were approached to enroll in the study declined participation. All subjects were consented prior to enrollment and the protocol followed the tenets of the Declaration of Helsinki; the study was approved by the Ethic Board of Zhongshan Ophthalmic Center.

We included all consecutive subjects with diagnosis of glaucoma, aged over 18 years old, self-administrating eye drops with no compliance aids more than 6 months, having better visual acuity no less than 20/200 in either eye, using at least one topical hypotensive medications in one or both eyes. Subjects were only excluded if they had disability in communication or physical impediments to eye drop use [5]. In addition, subjects were required to have had a complete ophthalmic exam within the preceding 6 months. If it was not available, the patient completed testing during study enrollment.

Subject Characteristics—we measured age as continuous variables and gender as a dichotomous variable. Levels of education was originally measured as a categorical variable and then was dichotomized for the multivariable analyses (i.e., under middle school, higher than middle school). The number of glaucoma medications being taken was measured as a continuous variable and then recoded into one versus two or more for the multivariable analyses. Length of time having using antiglaucoma eye drops was measured as a dichotomous variable: one year or more. Visual field defects were classified as mild (mean deviation ≥ −6 dB), moderate (mean deviation < −6 but > −12 dB), or severe (mean deviation ≤ −12 dB) according to Hodapp-Anderson criteria [1]. The reliability parameters of less than 20% errors were used.

Eye drop instillation technique—subjects were first escorted to an exam room and instructed to instill a 5 ml sterile artificial tear solution just as they usually did at home. They were free for a second attempt while they were not satisfied with their first attempt but no prompting was given. The right eye was assigned if the patients had prescribed eye drops for both eyes. The entire process was observed and recorded by two research staff. When there was disagreement between graders, a consensus grading was used. A comprehensive list of items associated with eye drop instillation techniques was developed, based on prior research [6]. Skill score system was based on previous studies [6, 7] which are demonstrated in Table 1. The total process patients instilled eye drops was recorded and scored on each eye drop attempt. Score of quite first drop and last drop, average score of skill of all eye drops, and total skill score were analyzed. Perfect instillation technique was defined as being to instill a single drop in the eye conjunctiva on the first attempt without touching one's eyelid or face. Participants were also asked to recall whether they had had any instruction on skills of instilling eye drops previously, and if so, from whom. Multiple linear regression was examined to explore how the demographic characteristics (gender, age, race, levels of education, number of glaucoma medications, and length of time diagnosed with glaucoma), clinical characteristics, knowledge of using eye drops, and instruction history were associated with total skill score. Next, in a different multivariable logistic regression model, we examined the same factors whether associated with the first drop score system (whether patients had successful perfect eye drop during the first try). Variables with a *p* < 0.05 in the univariable analysis were included in the multivariable regression model.

## 3. Results

One hundred and thirteen subjects participated in the study and their demographic characteristics are presented in Table 2. Sixty-two of the participates were male, 85% lived in urban and 60% had education level lower than middle school and subjects aged 56.5 years on average. Percentages of subjects with early, mild, and late stage of visual field defect severity for their better eyes were, respectively, 37.2%, 25.7%, and 37.2%. More than half of the sample (54.9%) had been using less than four eye drops and most (95.6%) had a history of less than one year in this study.

As shown in Table 3, most patients (93.8%) controlled the interval between eye drops more than 5 minutes; however, only 3.5% patients knew to press the dacryocyst area after instilling the eye drops. Only a small part of patients (9.7%) have training experience of using eye drops, of which mostly (81.8%) by doctor or nurse in the hospital.

From Table 4, only half (48.67%) of the participants finish administrating eye drops on the first try. The average drops used was 1.7 ± 0.8 which transformed to 4.35 ± 0.71 in total score system. The average total skill score was 6.93 ± 1.88 and first drop skill score was 0.04 ± 0.47. Of patients regarding quite first drop, 11 (11/113, 9.7%) missed the eye and got contamination with the drop (score of 0), 46 (46/113, 40.7%) missed the eye, 7 (7/113, 6.2%) got target but touched the tip of the bottle to the bulbar conjunctiva or cornea, and 24 (24/113, 21.2%) got target but touched the eyelid or lashes with the tip of the bottle. Twenty-five subjects (25/113, 22.1%) successfully finished administration during the first try, of which eight subjects (8/113, 7.1%) had the best expertise and scored highest.

Table 5 shows the results of the linear model for predicting good drop instillation technique. Total skill score was chosen as response variable. Factors of demographic, clinical characteristics, knowledge of using eye drops, and previous education were assessed by univariable analysis. It showed that younger age, better education, better visual acuity, knowledge of pressing the dacryocyst area to occlude the punctum, and prior instruction regarding using eye drops were the factors significantly associated with a good technique (*p* < 0.05 for all). In the multivariable model, only age, visual acuity, and previous education on drop instillation technique remained significant.  Table 6 shows the results of the logistic regression model for predicting whether it finished successfully in the first drop. Consistent with the total skill predicting model, same risk factors were confirmed in the univariable analysis. However, only age and visual acuity remained significant in multivariable regression. Prior instructions on using eye drops were not included in the final logistic regression model.

## 4. Discussion

Glaucoma is a slowly progressive eye disease, and prescribed glaucoma regimen adherence has long been an issue with glaucoma patients [5]. Improper administration of eye drops is often of a variety of unintentional noncompliance and underreported [8]. Unawareness is not only from patients but also from eye care providers especially in busy clinical practices [9]. Approximately 80% of patients instill their own eye drops by themselves and mostly, no delivery aids are adopted [10]. Our study indicates that only 19.7% patients managed to successfully instill eye drops into conjunctiva sac on first attempt.

Although volume of one drop (50 uL) dispensed from bottle far exceeds the capacity of conjunctiva sac (5 uL), our study showed it is not easy for patients to achieve the administration of medication. The mean drops squeezed by each patient per application per eye were 1.57. The mean drops instilled in the conjunctiva were 1.33. Brown et al. [11] found that 21% glaucoma patients administered 2 or more drops in their eyes by themselves. In a similar study, Dietlein et al. [12] reported that only 57% patients managed to instill eye drops in the conjunctival sac and 28.5% of patients closed their eyelids for at least 3 minutes after administration, while only 5.7% occluded the punctum. Even worse, only 3.5% (4/113) patients in our study tried to occlude the punctum after administration. A substantial amount of eye drops was wasted and increased the cost associated with treatment due to the faulty instillation technique. Unable to press the dacryocyst area again increased the bypass outflow of the medicine and systemic absorption. Unwanted adverse effects [13] includes those common side effects such as periocular hyperpigmentation caused by prostaglandin, arrhythmias associated with b-blockers, and infrequent side effects such as airway obstruction, thrombocytopenia, and electrolyte disturbances with carbonic anhydrase inhibitors. Adverse effects may bring frustration to patients, which decreases adherence, lowers the effectiveness, and therefore facilitates the swift of treatment to surgery.

Another issue studied in this study is the potential mechanical contact with ocular surface while instilling the eye drop. Underlying complications may range from infections of ocular surface or trauma such as corneal abrasions [14]. In developing countries, contamination to the bottle may happen due to bad living environment [15]. Dietlein et al. [12] reported that scratching of the eye drop container on the cornea or the conjunctiva was observed in 68% of the study group. Severe visual loss due to corneal ulcers caused by *Serratia marcescens*, secondary to the use of contaminated eye drop containers has been shown by Templeton et al. [16] in their study. Our study showed 37.7% patients got contamination by contacting with ocular surface or adnexa on their first attempt to instill the drops. It deserves great concern in clinical settings as the underlying devastating complications; also, the eye drops inherence such as instillation technique needs to be evaluated before changing the prescribed regime.

In this study, we investigated the associated determinants of the faulty techniques. Older age, worse visual acuity, and no instruction previously were found risk factor-associated faulty techniques. The patients in the study were aged on average 56.53 (range, 18 to 80), wherein the elderly population constitutes a major share as glaucoma mostly affected. Even though we excluded patients with physical inability for administrating eye drops, still older patients had reduced manual dexterity with age. Poor vision results in the inability of patients to visualize the tip of the bottle and erroneous judgments of the height of the tip of the bottle above the ocular plane. A lack of previous instruction obviously contributes to lack of knowledge about the correct technique, such as press the dacryocyst area and avoid the contamination of the drops. It should be an indispensable part of glaucoma care and further studies are needed to explore its positive effect on the adherence of glaucoma patients [17].

A limitation of this study should be noticed. Firstly, a confounding factor to be considered is that the eagerness of the patient to perform the task accurately when being observed by the study staff may also induce a behavioral modification. Secondly, we did not explore the difference of instillation technique in either eye when patients have prescribed eye drops for both eyes. Thirdly, we only included patients with better visual acuity no less than 20/200 in either eye as those with worse visual acuity may have companions to help. However, there are still a considerable number of patients who instill eye drops by themselves, which needs more attention in further studies. Still and all, our study again confirmed the reality of poor drop technique in glaucoma patients.

Despite the ongoing challenge of poor drug adherence of patients with glaucoma, potential strategies come up day in and day out to counter the issue of poor drop adherence. Smart reminding device, ocular sprays, drug-eluting contact lenses, and other modalities of all sorts are on the way to more patients [18]. Moreover, with the advent of surgical innovations, the minimally invasive surgery may have a much smaller chance of risk. Thus, the patients have more options rather than sticking to eye drops [19].

In conclusion, this study clearly shows that a vast majority of glaucoma patients in China are not correctly instilling eye drops. This can lead to serious consequences on the quality of life of the patients. It also highlights the importance of patient education with regard to eye drop instillation whenever glaucoma topical medications are prescribed and a check on this by the eye care providers during follow-up visits.

## Figures and Tables

**Table 1:** Scheme used to grade eye drop instillation technique.

Description of technique	Score
Good technique, on target, and no contamination	5
Awkward technique, on target, and no contamination	4
On target but contaminates by touching the bottle tip to the lashes or lid	3
On target but contaminates by touching the bottle tip to bulbar conjunctiva or cornea^∗^	2
Not on target, and no contamination	1
Not on target and contaminates the bottle tip by touching the eye, eyelid, or lashes	0

On target: delivered the eye drop to the eye or conjunctiva sac; ^∗^Added risk of trauma to ocular surface.

**Table 2:** Patients' demographic, clinical characteristics (*N* = 113).

Item	Result
*Demographic characteristics*	
Age (y), mean (SD)	56.53 (14.60)
Male sex, *n* (%)	62 (54.87)
Education level, *n* (%)	
≤middle school	60 (53.10)
>middle school	53 (46.90)
Location, *n* (%)	
Urban	85 (75.22)
Rural	28 (24.78)
*Clinical characteristics*	
UCVA, ^∗^* n* (%)	
Good (≥6/12)	84 (74.34)
Intermediate (<6/12 and ≥6/18)	10 (8.85)
Poor (<6/18)	19 (16.81)
Visual field stage, ^∗∗^* n* (%)	
Early stage	42 (37.17)
Medium stage	29 (25.66)
Late stage	42 (37.17)
Number of local eye drops, *n* (%)	
<4	62 (54.87)
4–6	51 (45.13)
Duration of using eye drops (y), *n* (%)	
<1	108 (95.58)
1–5	5 (4.42)

SD: standard deviation; UCVA: uncorrected visual acuity. ^∗^Use UCVA of the eye, which had better visual acuity in this item. ^∗∗^Use visual field of the eye, which had better visual acuity in this item.

**Table 3:** Patients' knowledge and education experience of using eye drops (*N* = 113).

Item	Result
*Knowledge of using eye drops*	
Interval between each eye drops, *n* (%)	
5 minutes	7 (6.19)
10 minutes	105 (92.92)
30 minutes	1 (0.88)
Press dacryocyst area, *n* (%)	
Yes	4 (3.54)
No	109 (96.46)
*Education/training experience of using eye drops*	
Having training of using eye drops, *n* (%)	
Yes	11 (9.73)
No	102 (90.27)
Education method, *n* (%)	
By doctor/nurse in ZOC	8 (72.73)
By doctor/nurse in other hospital	1 (9.09)
Reading education brochure	2 (18.18)
Education time, *n* (%)	
5 minutes	4 (36.36)
10 minutes	5 (45.45)
30 minutes	2 (18.18)

SD: standard deviation; ZOC: Zhongshan Ophthalmic Center.

**Table 4:** Patients' skill of using eye drops.

Item	Result
*Skill score system*	
Score of quite first drop of eye drops (0.5), mean (SD)	0.04 (0.47)
Score of number of last drop (0–5), mean (SD)	4.35 (0.71)
Average score of skill of each eye drops (0–5), mean (SD)	2.53 (1.15)
Total skill score (0–15), mean (SD)	6.93 (1.88)
*First drop score system*	
Finish in first drop, *n* (%)	
Yes	55 (48.67)
No	58 (51.33)

SD: standard deviation.

**Table 5:** Linear model of potential predictors of patients' skill score of using eye drops (skill score system) (significant items were highlighted in bold).

	Simple regression		Multiple regression^†^	
	*β* (95% CI)	*p* value	*β* (95% CI)	*p* value
*Demographic characteristics*				
Mean of age	**−0.05 (−0.07, −0.03)**	**<0.001**	**−0.03 (−0.05, −0.01)**	**0.002**
Male sex	−0.56 (−1.26, 0.14)	0.118		
Education level				
≤middle school	Reference			
>middle school	**0.88 (0.19, 1.56)**	**0.013**	0.51 (−0.10, 1.11)	0.100
Location				
Urban	Reference			
Rural	0.31 (−0.51, 1.13)	0.453		
*Clinical characteristics*				
UCVA^∗^				
Good (≥6/12)	Reference			
Intermediate (<6/12 and ≥6/18)	0.50 (−0.71, 1.72)	0.416	0.10 (−0.94, 1.15)	0.843
Poor (<6/18)	**−1.25 (−2.18, −0.33)**	**0.008**	**−1.24 (−2.03, −0.45)**	**0.002**
Visual field stage^∗∗^				
Early stage	Reference			
Medium stage	−0.61 (−1.51, 0.28)	0.179		
Late stage	0.22 (−0.59, 1.03)	0.588		
Number of local eye drops				
<4	Reference			
≥4	0.40 (−0.30, 1.11)	0.263		
Duration of using eye drops (y)				
<1	Reference			
1–5	0.70 (−1.01, 2.41)	0.421		
*Knowledge of using eye drops*				
Interval between each eye drops				
5 minutes	Reference			
10 minutes	−0.78 (−2.23, 0.67)	0.289		
30 minutes	−3.69 (−7.66, 0.28)	0.068		
Press dacryocyst area				
Yes	**3.70 (1.92, 5.48)**	**<0.001**	1.64 (−0.12, 3.40)	0.068
No	Reference			
*Education/training experience of using eye drops*				
Having training of using eye drops				
Yes	**2.54 (1.45, 3.63)**	**<0.001**	**1.71 (0.62, 2.80)**	**0.002**
No	Reference			

SD: standard deviation; UCVA: uncorrected visual acuity. ^∗^Use patient better-seeing eye's UCVA in this item. ^∗∗^Use patient better-seeing eye's visual field in this item. ^†^All variables in the simple regression with *p* < 0.05 were included in multiple regression.

**Table 6:** Logistic model of potential predictors of patients' skill score of using eye drops (first drop score system) (significant items were highlighted in bold).

	Simple regression		Multiple regression^†^	
	*β* (95% CI)	*p* value	*β* (95% CI)	*p* value
*Demographic characteristics*				
Mean of age	**−0.01 (−0.02, −0.004)**	**0.002**	**−0.01 (−0.01, −0.002)**	**0.010**
Male sex	0.14 (−0.05, 3.24)	0.151		
Education level				
≤middle school	Reference			
>middle school	0.15 (−0.04, 0.34)	0.115		
Location				
Urban	Reference			
Rural	0.07 (−0.15, 0.28)			
*Clinical characteristics*				
UCVA^∗^				
Good (≥6/18)	Reference			
Intermediate	0.52(−0.27, 0.37)	0.747	−0.01 (−0.32, 0.30)	0.962
Poor (<6/18)	**−0.39 (−0.63, −0.15)**	**0.002**	**−0.38 (−0.61, −0.14)**	**0.002**
Visual field stage^∗∗^				
Early stage	Reference			
Medium stage	−0.17 (−0.41, 0.07)	0.168		
Late stage	−0.05 (−0.26, 0.17)	0.665		
Number of local eye drops				
<4	Reference			
≥4	0.08 (−0.11, 0.27)	0.415		
Duration of using eye drops (y)				
<1	Reference			
1–5	−0.09 (−0.55, 0.37)	0.695		
*Knowledge of using eye drops*				
Interval between each eye drops				
5 minutes	Reference			
10 minutes	0.07 (−0.32, 0.46)	0.735		
30 minutes	−0.43(−1.50, 0.64)	0.428		
Press dacryocyst area				
Yes	**0.53 (0.03, 1.04)**	**0.037**	0.16 (−0.36, 0.68)	0.542
No	Reference			
*Education/training experience of using eye drops*				
Having training of using eye drops				
Yes	**0.37 (0.06, 0.68)**	**0.020**	0.26 (−0.06, 0.58)	0.109
No	Reference			

SD: standard deviation; UCVA: uncorrected visual acuity. ^∗^Use patient better-seeing eye's UCVA in this item. ^∗∗^Use patient better-seeing eye's visual field in this item. ^†^All variables in the simple regression with *p* < 0.05 were included in multiple regression.
